# Genotypes-Independent Optimization of Nitrogen Supply for Isolated Microspore Cultures in Barley

**DOI:** 10.1155/2016/1801646

**Published:** 2016-07-25

**Authors:** Ruiju Lu, Zhiwei Chen, Runhong Gao, Ting He, Yifei Wang, Hongwei Xu, Guimei Guo, Yingbo Li, Chenghong Liu, Jianhua Huang

**Affiliations:** ^1^Biotech Research Institute, Shanghai Academy of Agricultural Sciences, Shanghai 201106, China; ^2^Shanghai Key Laboratory of Agricultural Genetics and Breeding, Shanghai 201106, China

## Abstract

To establish a high-efficiency system of isolated microspore culture for different barley genotypes, we investigated the effects of nitrogen sources and concentrations on callus induction and plant regeneration in different barley genotypes. The results showed that the organic nitrogen sources greatly increased the callus induction, and the great reduction of total nitrogen sources would significantly decrease the callus induction. And the further optimization experiments revealed that the increasing of organic nitrogen sources was much important in callus induction while it seemed different in plant regeneration. Based on the great effects of organic nitrogen on callus induction, the medium of N6-ANO1/4-2000 might be the best choice for the microspore culture system. In addition, the phylogenetic analysis indicated that there were clear differences of genetic backgrounds among these barley genotypes, and it also suggested that this medium for microspore culture had widespread utilization in different barley genotypes.

## 1. Introduction

A haploid plant contains a single set of chromosomes normally from a gamete in diploid plants. Haploids can be easily induced to homozygous double haploids (DHs) in natural chromosome doubling or by artificial chromosome doubling with the colchicine. The DH technology has many applications in plant breeding and genetic researches. It can be used to improve breeding efficiencies by stabling genetic variations quickly, especially the selection on traits controlled by recessive genes [1, 2]. And it is an excellent tool for using in the researches of genetic transformation and mutagenesis [3]. It also can be used in genetic studies such as constructions of DHs populations for QTL mapping in many crops [4], and haploids or DHs have recently been thought as ideal sources of genomic DNA for genome sequencing because of their pure DNA backgrounds. In addition, it provides a unique tool for investigation of the microspore embryogenesis in vitro [5, 6].

So far, the anther culture and the microspore culture have been thought as the main methods for producing haploids. And the anther culture could be interfered by heterozygous diploid somatic cells from anther wall tissues, while the microspore culture system is an ideal way to produce pure haploids or DHs. Moreover, the microspore culture has higher efficiency of potential to produce embryoids and then green haploid plants. Davies and Morton [7] found that microspore culture system produced 5~200-fold more green haploid plants than the anther culture system in barley under optimized conditions. A highly efficient haploids production system is critical for the application of this technology.

Since the first report of haploid plants formations by isolated microspore culture in tobacco and Datura [8], the haploid plants regenerations by isolated microspore culture then have been reported in many crops including wheat, barley, rice, and oilseed rape [9]. Although there is an efficient system for green haploid plants regenerations by isolated microspore culture in tobacco [9], situations are different in other plant species [5]. It is because the fact that these processes are influenced by many factors, including the internal factors (e.g., genotype and developmental stage of the microspores) and external factors (e.g., growth environment of donor plant, pretreatment, and culture medium) [10]. Considering the general use of microspore culture in different plant genotypes, high efficiency of plant regeneration can be also achieved through optimization of medium compositions [11–14].

Manipulation of medium compositions, like macronutrients (carbon and nitrogen sources) and micronutrients (e.g., copper and zinc), has been proven effectively for improving plant regeneration [15–19]. In barley, early researches showed that the decreases of ammonia nitrate level in the induction medium were beneficial in anther culture [20–22]. And the plant regeneration could be significantly increased by a combination of ammonia nitrate reduction and glutamine supplement [21]. Mordhorst and Lörz [23] found the importance of nitrogen sources for the initiation of androgenesis and plant regeneration in isolated microspore culture of barley. All these researches resulted in significant advances in anther culture and microspore culture of Igri, while they were highly affected by the growth environments and the donor plants themselves [24, 25]. Therefore, further researches are also required to develop higher efficient microspore culture systems in barley and to create more haploid green plants. As we know, SSR markers are useful tools for discriminating different barley genotypes [26]. And this would make sure that we used different barley genotypes with different genetic backgrounds. In this study, we attempted to minimize effects of genotypes on isolated microspore culture by using different barley genotypes and manipulation of nitrogen supply in the culture medium.

## 2. Materials and Methods

### 2.1. Materials

Donor plants of barley genotypes of Hua-11, Hua-22, Hua-30, BR06-5, BI-04, BI-06, BI-28, BI-32, BI-45, BI-49, and BI-55, which were collected from different areas of China, were grown in experimental fields of Shanghai in November 2011. The plans were normally managed and inflorescences were collected in April 2012. Tillers of Hua-11, Hua-22, Hua-30, BI-04, BI-06, BI-28, BI-45, and BI-55 were collected and stored in refrigerator at 4°C temperately for later microspore isolation or DNA extraction.

### 2.2. Preparation of the Microspore Isolation Buffer and Culture Mediums

Microspore isolation buffer contained 60 g/L mannitol, 1.1 g/L CaCl_2_, and 0.976 g/L MES. The induction media were referred to N6 basal medium by some modifications [27] (Table 1). Both buffer and culture mediums were adjusted to pH 5.8 and then sterilized by filter-sterilization. The differentiation medium was MS-based [28] and supplemented with kinetin 1.5 mg/L, 6-BA 0.5 mg/L, and NAA 0.01 mg/L, with 30 g/L maltose as carbon supply and solidified with agar at 6 g/L. This medium was sterilized by autoclave sterilization (0.11 Mpa, 121°C for 15 minutes).

### 2.3. Callus Introduction and Differentiation

Microspores were isolated as described by Lu et al. [29]. Isolated microspores were rinsed once with the induction medium and adjusted to a density of 5.0 × 10^5^ microspores/mL medium. Each volume of 1 mL of this microspore mixture was then transferred into petri dishes (size of 35 mm × 15 mm), sealed with parafilm, and incubated in darkness at 25°C for about 28 days, four replicates for each sample. While for the plant regeneration, the microspore culture for induction of callus was in darkness for 21 days, the calli were transferred into new petri dishes within differentiation medium for plant regeneration at 25 under light of 150 *μ*mol m^−2^s^−1^ with 16 h photoperiod.

### 2.4. Measuring of the Callus Yields and Green Plants

The yields of calli were directly measured by weighing the total calli formed in each petri dish after induction for 28 or 21 days. The green plants were directly counted after induction on differentiation medium from three weeks to two months, and the green plant rate was the number of green plants outputs from each 100 mg calli.

### 2.5. DNA Extraction and SSR Marker Analysis

The DNA extraction was carried out by using Ezup pillar type plant genomic DNA extraction kit (Sangon Biotech, China); then the DNA quality and concentration were detected by using NanoDrop ND-100. 11 SSR markers were chosen and used for SSR marker analysis according to Chen et al. [26] with some modifications of gel running (120 v for 2 h) and staining (by using SYBR Gold Nucleic Acid Gel Stain).

### 2.6. Data Analysis

All general data were analyzed by using MS excel software; the statistics were conducted by SPSS of version 22. The methods were used for the calculation of polymorphism information content (PIC) according to Anderson et al. [30]. The dendrogram was constructed by using the methods according to Sneath and Sokal [31] and NTSYS-pc version 2.10 package [32].

## 3. Results

### 3.1. Effects of Nitrogen Sources on Callus Induction

Four barley genotypes (BI-06, BI45, Hua-30, and BR06-5) were used for investigation of the effects of different nitrogen sources on callus induction in microspore culture. It was showed that the callus yields were significantly affected by both different nitrogen sources and different barley genotypes (Table 2). Callus yields were the lowest when only one inorganic nitrogen source was used, KNO_3_ or (NH_4_)_2_SO_4_, and the callus yields of them were 50.5 mg/dish and 56.8 mg/dish in average, respectively (Table 3). Both of KNO_3_ and (NH_4_)_2_SO_4_ were together used as nitrogen sources which resulted in a significant increase in callus yields by 94.3 mg/dish in average (Table 3). In addition, the callus yields would be increased further if using organic nitrogen sources, and the callus yields of BI-06 and BI-45 peaked in medium of N6-AO, Hua-30 peaked in N6-O, and BR06-5 peaked in N6-ANO. And they are significantly higher than those in mediums within inorganic nitrogen sources only. Therefore, two kinds of inorganic nitrogen sources are better than single one for callus induction, and organic nitrogen sources will further increase callus induction.

### 3.2. Effects of Different Nitrogen Concentrations on Callus Induction

Five barley genotypes were used to examine the effect of nitrogen concentrations on callus induction, including BI-06, BI-28, BI-32, BI-45, and BI-49.  Table 4 showed that there were significant differences of callus yields both in different nitrogen concentrations and in different barley genotypes. Based on the medium of N6-ANO, serious reductions of callus yields occurred in N6 without any additional nitrogen sources, while no significant reductions occurred in N6-ANO3/4 (Table 5). And there was only BI-49 which had a significant reduction from N6-ANO1/2 (while there was no significant difference between N6-ANO1/2 and N6-ANO1/4 in BI-49), and BI-32 had a significant reduction from N6-ANO1/4 (Table 5). And the callus yields of BI-28 and BI-32 peaked in N6-ANO3/4, and BI-06 and BI-45 peaked in ANO1/2, although there were no significant differences comparing to N6-ANO (Table 5). Thus, it suggested that simultaneous decreasing of both inorganic nitrogen sources and organic nitrogen sources at certain extents had no significant effects on callus induction, while big decreasing of these nitrogen sources together would decrease callus induction significantly. Considering the different roles of inorganic nitrogen sources and organic nitrogen sources, decreasing inorganic nitrogen sources while increasing organic nitrogen source should be better for callus induction.

### 3.3. Effects of Different Organic Nitrogen Concentrations on Callus Induction and Differentiation

Based on the previous results, we further investigated the effects of decreased inorganic nitrogen sources while increasing organic nitrogen sources on callus induction and green plants differentiation together in eight different barley genotypes based on the medium of N6-ANO.

**Figure 1:**
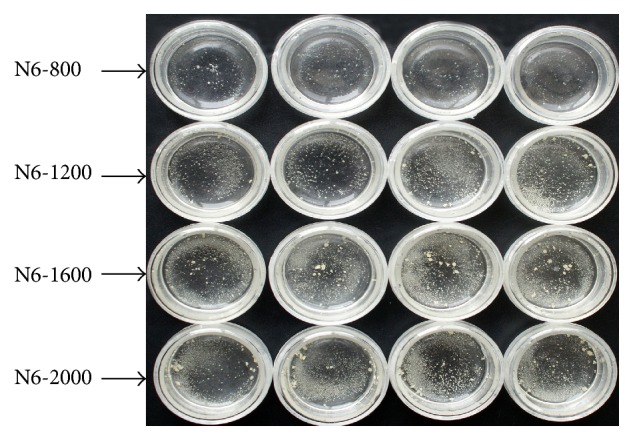
Callus induction of Hua-11 in media within different concentrations of organic nitrogen sources. Four dishes of each treatment represent four replicates.

Table 6 showed that there were significant differences of callus yields both in different organic nitrogen concentrations and in different barley genotypes. And the callus yields of all barley genotypes increased with the increasing of organic nitrogen in medium (Table 7, Figure 1), and they all peaked under the medium of N6-ANO1/4-2000 except BI-45 (while the highest callus yields of BI-45 under the medium of N6-ANO1/4-1600 had no significant differences to those under the medium of N6-ANO1/4-2000). In addition, the medium of N6-ANO1/4-800 just decreased the inorganic nitrogen sources comparing to the medium of N6-ANO, while the callus yields under N6-ANO1/4-800 were higher than those under N6-ANO except the genotype of BI-45 (although there was no significant differences between them). It suggested that decreasing inorganic nitrogen sources while increasing organic nitrogen sources in medium was beneficial for callus induction, and the medium of N6-ANO1/4-2000 should be the best one for callus induction.

Table 8 showed that there were significant differences of green plant rates both in different organic nitrogen concentrations and in different barley genotypes. While the multiple comparing showed that there were only two genotypes (BI-06 and BI-55) which were significantly affected by organic nitrogen concentrations, the green plant rates seemed to decrease with the increasing of organic nitrogen concentrations (Table 9). And BI-06 exhibited a significant decrease till in medium of N6-ANO1/4-2000, while BI-55 was from the medium of N6-ANO1/4-1200 (Table 9). With further comparing the values of green plant rates among different media, the effects of organic nitrogen on green plant regeneration were smaller than those on callus induction. Therefore, the medium of N6-ANO1/4-2000 should be considered for much higher increasing of callus induction that would compensate the smaller reduction of green plant rate.

### 3.4. Phylogenetic Analysis of the 8 Barley Genotypes

11 SSR markers were chosen for SSR analysis, and 6 of them with clear and steady bands were used for statistics among 8 barley genotypes. A total of 44 alleles were observed at the 6 SSR loci, ranging from 4 to 9 with an average of 7.3 (Table 6), and PIC values ranged from 0.691 to 0.875 with an average of 0.818 (Table 10).

**Figure 2:**
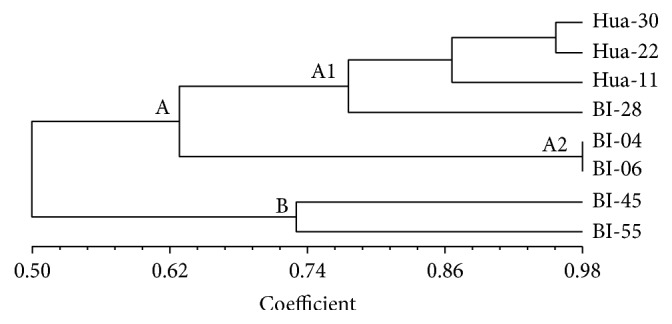
Dendrogram showing the genetic relationship of 8 barley genotypes based on SSR markers.

The dendrogram (Figure 2), which was obtained by using the SM coefficient, consisted of two main categories (A and B). The A category was further divided into two subgroups (A1 and A2). Considering these barley genotypes themselves, Hua-30, Hua-22, and Hua-11 are from Shanghai area of China, BI-04 and BI-06 are from the north of Jiangsu province of China, BI-28 is from Henan province of China, and BI-45 and BI-55 are from Heilongjiang province of China. So these barley genotypes used in this study had the representativeness of various barley production areas in China. And from the clustering analysis, we found that barley genotypes from the north China plain and the Middle-Lower Yangtze Plain were closer than those from the northeast China plain in genetically. Interestingly, Hua-30, Hua-22, Hua-11, and BI-28 were closer than BI-04 and BI-06. While for the further investigation, we found that BI-06 was introduced from Japan, and this might be the reason of the difference.

## 4. Discussions

Nitrogen supply in terms of the quantity and ratio of nitrate, ammonia, and amino acids in induction media played a critical role in the development of plant anther and isolated microspore culture. Clapham [20] generated pollen plants for the first time after reducing ammonia nitrate content by 10-fold (from 20 mM to 2 mM) in MS-based induction medium. In tobacco, glutamine (5.5 mM) and serine (1 mM) were found to promote embryoid development in microspore culture [8]. Glutamine was also shown to be beneficial for growth and development of somatic embryoids in cell suspension cultures of carrot [33]. And barley anther culture was improved by reducing nitrate from 20 mM to 0.2 mM while supplementing glutamine (optimal level of 5.1 mM) in the induction medium [21]. Thus, decreasing inorganic nitrogen while increasing organic nitrogen sources might be better for microspore culture based green plant regeneration. And our study confirmed the benefits of organic nitrogen especially in callus induction though there were some barley genotype-based differences, and the callus induction was crucial for the green plant regeneration at last. Although most researchers found that there were clear beneficial effects of organic nitrogen such as glutamine for anther and microspore culture, the negative effects of glutamine were also reported on anther and microspore culture of barley [17, 23, 34, 35]. And we also observed that there were controversial effects of organic nitrogen on callus differentiation or green plant regeneration in some barley genotypes, though most of barley genotypes were not affected by changing organic nitrogen sources. Anyhow, it indicated that the responses to nitrogen on callus induction were different on green plant regeneration, and this was consistent with an earlier report [36].

Mordhorst and Lörz [23] made a more detailed research about nitrogen sources on microspore culture, and they found that the optimal nitrogen supply for their barley isolated microspore culture was 20~35 mM of total nitrogen, and there were more irregularly shaped aggregates if there is too much supply of nitrogen while the embryo formation would be repressed if there is too low supply of nitrogen, and the ratio of nitrate to ammonia was best at 90 : 10, and single inorganic nitrogen or too high of ammonia would decrease the plating efficiency, and the ratio of organic nitrogen to inorganic nitrogen was better at 71 : 29, and higher organic nitrogen would decrease green plant regeneration. In our study, we found that appropriate reduction of nitrogen concentrations in culture media did not affect callus induction though there were genotype-based differences of reduction extents, while too low nitrogen concentration would significantly inhibit the callus induction. And there was no significant differences when using single nitrogen source of nitrate or ammonia in callus induction in our study, while using both of the two inorganic nitrogen sources significantly increases the callus induction except the barley genotype of Hua 30; this also showed that the use of nitrate and ammonia together would be beneficial for callus induction, while we did not find the differences between nitrate and ammonia. Like previous discussion of negative effects of organic nitrogen on green plant regeneration, it was not common in our study although it happened in some barley genotypes.

In this study, we tried to use liquid culture media to collect calli as much as possible, and we also found that the callus induction played a crucial role for production of green plants although supplement of organic nitrogen sources in the media had different effects between callus induction and green plant regeneration. And supplementing organic nitrogen sources in media within decreased inorganic nitrogen sources significantly increased callus yields and then obtained more green plants in callus differentiation, and this also suggested that manipulating nitrogen supply in media of microspore culture might be an effective way to improve callus induction and green plant regeneration. Here, we suggested that the media of N6-ANO1/4-2000 should be the best choice for callus induction and green plant regeneration in microspore culture of barley, or we could use the media within lower organic nitrogen sources for green plant regeneration.

In addition, we did a genetic analysis of barley genotypes by SSR markers, and it showed that there were obvious genetic differences among these barley genotypes and the classification by genetic markers was consistent with their distributions. And we also found that it was difficult to discriminate barley genotype of BI-04 and BI-06 by genetic markers though they were quite different in response to nitrogen on callus induction and green plant regeneration. So it might be that less markers used in this study were not enough to divide them for they had parts of same genetic backgrounds.

## Figures and Tables

**Table 1:** Culture media within different nitrogen sources.

Induction media	Notes	Ammonia nitrogen (NH_4_)_2_SO_4_ (mg/L)	Nitrate nitrogen KNO_3_ (mg/L)	Organic nitrogen	
				Glutamine (mg/L)	Casein hydrolysate (mg/L)
N6-ANO	N6 medium supplemented with ammonia, nitrate, and organic nitrogen sources	463.0	2830.0	800	800

N6-A	N6 medium supplemented only with ammonia nitrogen	463.0	0	0	0

N6-N	N6 medium supplemented only with nitrate nitrogen	0	2830.0	0	0

N6-O	N6 medium supplemented with two organic nitrogen sources	0	0	800	800

N6-AN	N6 medium supplemented with both ammonia and nitrate	463.0	2830.0	0	0

N6-AO	N6 medium supplemented with ammonia and two organic nitrogen sources	463.0	0	800	800

N6-NO	N6 medium supplemented with nitrate and two organic nitrogen sources	0	2830.0	800	800

N6-ANO3/4	N6 medium supplemented with 3/4 of nitrogen sources of N6-ANO	347.3	2122.5	600	600

N6-ANO1/2	N6 medium supplemented with 1/2 of nitrogen sources of N6-ANO	231.5	1415.0	400	400

N6-ANO1/4	N6 medium supplemented with 1/4 of nitrogen sources of N6-ANO	115.8	707.5	200	200

N6	N6 medium supplemented without any nitrogen	0	0	0	0

N6-ANO1/4-2000	N6 medium supplemented with 1/4 levels of inorganic nitrogen sources of N6-ANO, plus 2000 mg of each organic nitrogen	115.8	707.5	2000	2000

N6-ANO1/4-1600	N6 medium supplemented with 1/4 levels of inorganic nitrogen sources of N6-ANO, plus 1600 mg of each organic nitrogen	115.8	707.5	1600	1600

N6-ANO1/4-1200	N6 medium supplemented with 1/4 levels of inorganic nitrogen sources of N6-ANO, plus 1200 mg of each organic nitrogen	115.8	707.5	1200	1200

N6-ANO1/4-800	N6 medium supplemented with 1/4 levels of inorganic nitrogen sources of N6-ANO, plus 800 mg of each organic nitrogen	115.8	707.5	800	800

**Table 2:** Two-way ANOVA analysis of callus yields of different barley genotypes in media within different nitrogen sources.

Factor	d.f.	SS	MS	*F*-ratio	*P* value
Genotype	3	3247.7	1082.6	7.21	<0.001
Nitrogen source	6	157137.8	26189.6	174.41	<0.001
Interaction	18	10892.5	605.1	4.03	<0.001
Error	84	12613.3	150.2		

**Table 3:** Callus yields (mg/petri dish) in media of different nitrogen sources and significant difference analysis among different media in each barley genotype.

Genotype	Medium						
	N6-A	N6-N	N6-AN	N6-O	N6-ANO	N6-AO	N6-NO
BI-06	50.5 ± 3.8^a^	56.8 ± 16.0^a^	94.3 ± 1.9^b^	167.0 ± 4.2^c^	122.0 ± 11.4^bd^	145.8 ± 8.9^cd^	142.0 ± 24.3^cd^
BI-45	47.8 ± 11.8^a^	49.3 ± 1.5^a^	77.0 ± 9.7^b^	120.8 ± 10.1^c^	131.3 ± 4.4^c^	138.3 ± 11.2^c^	118.0 ± 14.0^c^
Hua-30	49.0 ± 14.7^a^	63.8 ± 1.7^a^	86.0 ± 9.8^a^	158.0 ± 15.5^b^	121.3 ± 31.3^ab^	151.0 ± 17.1^b^	129.5 ± 17.4^b^
BR06-5	44.0 ± 4.2^a^	48.0 ± 0.8^a^	105.5 ± 5.3^b^	119.0 ± 12.1^bd^	147.0 ± 5.4^c^	134.0 ± 4.2^cd^	115.3 ± 8.3^b^

Note: data were presented as mean ± standard deviation (*n* = 4). The different letters in the same line mean significant difference by Tukey's HSD (*P* < 0.05).

**Table 4:** Two-way ANOVA analysis of callus yields of different barley genotypes in media within different nitrogen concentrations.

Factor	d.f.	SS	MS	*F*-ratio	*P* value
Genotype	4	35128.7	8782.2	54.66	<0.001
Nitrogen concentration	4	97133.5	24283.4	151.14	<0.001
Interaction	16	21676.8	1354.8	8.43	<0.001
Error	75	12050.0	160.7		

**Table 5:** Callus yields (mg/petri dish) in media within different nitrogen concentrations and significant difference analysis among different media in each barley genotype.

Genotype	Medium				
	N6-ANO	N6-ANO3/4	N6-ANO1/2	N6-ANO1/4	N6
BI-06	122.0 ± 11.4^a^	121.5 ± 13.2^a^	127.8 ± 17.1^a^	124.8 ± 7.6^a^	54.5 ± 5.5^b^
BI-28	73.0 ± 8.8^a^	84.8 ± 7.5^a^	79.5 ± 10.5^a^	73.5 ± 7.2^a^	52.5 ± 3.5^b^
BI-32	135.5 ± 12.5^a^	147.0 ± 30.6^a^	133.0 ± 5.7^a^	93.3 ± 2.5^b^	31.5 ± 4.4^c^
BI-45	131.3 ± 4.4^ab^	155.0 ± 22.0^ab^	159.3 ± 20.1^a^	125.5 ± 6.1^b^	50.5 ± 3.1^c^
BI-49	168.0 ± 18.7^a^	159.8 ± 17.2^a^	129.5 ± 15.5^b^	106.8 ± 5.0^b^	53.0 ± 2.9^c^

Note: data were presented as mean ± standard deviation (*n* = 4). The different letters in the same line mean significant difference by Tukey's HSD (*P* < 0.05).

**Table 6:** Two-way ANOVA analysis of callus yields of different barley genotypes in media within different concentrations of organic nitrogen sources.

Factor	d.f.	SS	MS	*F*-ratio	*P* value
Genotype	7	237622.3	33946.0	375.07	<0.001
Nitrogen source	4	137896.2	34474.0	380.90	<0.001
Interaction	28	30055.1	1073.4	11.86	<0.001
Error	120	10860.8	90.5		

**Table 7:** Callus yields (mg/petri dish) in media within different concentrations of organic nitrogen sources and significant difference analysis among different media in each barley genotype.

Genotype	Medium				
	N6-ANO	N6-ANO1/4-800	N6-ANO1/4-1200	N6-ANO1/4-1600	N6-ANO1/4-2000
BI-04	17.0 ± 3.0^a^	59.2 ± 14.7^a^	92.0 ± 9.7^b^	102.4 ± 6.2^b^	108.0 ± 3.8^b^
BI-06	123.4 ± 9.7^a^	132.4 ± 13.0^a^	147.3 ± 6.1^a^	198.0 ± 22.3^b^	253.3 ± 15.7^c^
BI-28	19.1 ± 5.2^a^	37.7 ± 8.6^a^	42.0 ± 6.1^ab^	54.5 ± 10.5^bc^	68.5 ± 4.1^c^
BI-45	119.4 ± 3.0^a^	118.9 ± 9.9^a^	138.9 ± 8.1^ab^	157.2 ± 15.5^b^	157.1 ± 11.2^b^
BI-55	90.5 ± 0.6^a^	110.6 ± 4.0^a^	118.0 ± 3.4^a^	153.2 ± 18.8^b^	159.2 ± 3.4^b^
Hua-11	14.1 ± 4.2^a^	41.8 ± 5.0^a^	100.1 ± 6.1^b^	115.0 ± 4.6^c^	126.2 ± 6.0^c^
Hua-22	99.9 ± 9.4^a^	114.4 ± 7.0^a^	129.8 ± 4.1^ab^	149.9 ± 12.0^bc^	157.9 ± 12.1^c^
Hua-30	74.7 ± 13.0^a^	103.7 ± 4.8^a^	125.7 ± 2.3^b^	165.5 ± 15.0^c^	166.2 ± 2.3^c^

Note: data were presented as mean ± standard deviation (*n* = 4). The different letters in the same line mean significant difference by Tukey's HSD (*P* < 0.05).

**Table 8:** Two-way analysis of green plant rates of different barley genotypes in media within different concentrations of organic nitrogen sources.

Factor	d.f.	SS	MS	*F*-ratio	*P* value
Genotype	7	480891.4	68698.8	792.73	<0.001
Nitrogen source	4	1382.2	345.5	3.99	0.005
Interaction	28	3081.5	110.1	1.27	0.189
Error	120	10399.4	86.7		

**Table 9:** Green plant rates (green plants/100 mg calli) in media within different concentrations of organic nitrogen sources and significant difference analysis in different media in each barley genotype.

Genotype	Medium				
	N6-ANO	N6-ANO1/4-800	N6-ANO1/4-1200	N6-ANO1/4-1600	N6-ANO1/4-2000
BI-04	132.2 ± 7.3^a^	136.5 ± 24.2^a^	160.1 ± 27.7^a^	144.7 ± 18.6^a^	129.9 ± 7.0^a^
BI-06	9.1 ± 0.6^a^	8.9 ± 1.9^a^	7.8 ± 0.9^ab^	6.5 ± 1.6^ab^	5.2 ± 0.8^b^
BI-28	64.2 ± 4.1^a^	63.3 ± 10.1^a^	61.5 ± 7.9^a^	61.2 ± 6.3^a^	60.6 ± 9.4^a^
BI-45	2.9 ± 0.4^a^	3.4 ± 1.2^a^	3.4 ± 0.7^a^	2.6 ± 1.2^a^	2.4 ± 0.7^a^
BI-55	22.1 ± 0.9^a^	19.3 ± 4.2^ab^	14.8 ± 1.1^b^	8.8 ± 1.5^c^	8.1 ± 1.7^c^
Hua-11	61.6 ± 4.7^a^	62.5 ± 4.9^a^	64.5 ± 8.4^a^	61.6 ± 5.2^a^	58.9 ± 3.0^a^
Hua-22	151.5 ± 18.0^a^	156.9 ± 10.5^a^	156.8 ± 13.8^a^	141.2 ± 15.1^a^	138.9 ± 3.3^a^
Hua-30	101.8 ± 8.2^a^	108.6 ± 7.6^a^	106.6 ± 10.9^a^	106.2 ± 6.1^a^	102.6 ± 5.3^a^

Note: data were presented as mean ± standard deviation (*n* = 4). The different letters in the same line mean significant difference by Tukey's HSD (*P* < 0.05).

**Table 10:** Polymorphic SSR markers and the polymorphism information content (PIC).

SSR marker	Chr.	Total alleles	Polymorphic alleles	PIC
Bmac213	1	8	8	0.864
Bmac0134	2	8	5	0.855
EBmac679	4	9	9	0.875
Bmag500	6	8	7	0.834
Bmac602	6	7	5	0.790
GBM1404	6	4	1	0.691

Mean		7.3	5.8	0.818
